# VGSC: A Web-Based Vector Graph Toolkit of Genome Synteny and Collinearity

**DOI:** 10.1155/2016/7823429

**Published:** 2016-02-24

**Authors:** Yiqing Xu, Changwei Bi, Guoxin Wu, Suyun Wei, Xiaogang Dai, Tongming Yin, Ning Ye

**Affiliations:** ^1^School of Computer Science and Engineering, Southeast University, Nanjing, Jiangsu 211189, China; ^2^The Southern Modern Forestry Collaborative Innovation Center, Nanjing Forestry University, Nanjing, Jiangsu 210037, China; ^3^College of Information Science and Technology, Nanjing Forestry University, Nanjing, Jiangsu 210037, China; ^4^College of Forest Resources and Environment, Nanjing Forestry University, Nanjing, Jiangsu 210037, China

## Abstract

*Background*. In order to understand the colocalization of genetic loci amongst species, synteny and collinearity analysis is a frequent task in comparative genomics research. However many analysis software packages are not effective in visualizing results. Problems include lack of graphic visualization, simple representation, or inextensible format of outputs. Moreover, higher throughput sequencing technology requires higher resolution image output.* Implementation*. To fill this gap, this paper publishes VGSC, the Vector Graph toolkit of genome Synteny and Collinearity, and its online service, to visualize the synteny and collinearity in the common graphical format, including both raster (JPEG, Bitmap, and PNG) and vector graphic (SVG, EPS, and PDF).* Result*. Users can upload sequence alignments from blast and collinearity relationship from the synteny analysis tools. The website can generate the vector or raster graphical results automatically. We also provide a java-based bytecode binary to enable the command-line execution.

## 1. Introduction

Synteny is the collection of contiguous genes located on the chromosome of different species. Collinearity is a particular kind of synteny in which the genes are conserved in the same order [1]. Understanding this colocalization of genetic loci amongst species is a frequent task in comparative genomics research, and it often relies on the accuracy of homology identification within or across genomes. During the evolution, eukaryotic genomes between different species reveal this synteny and collinearity in various levels [2]. There are many reasons for the structural variation of genes all over the long evolutionary history, such as whole-genome duplication (WGD), segmental duplication, inversions, and translocations [3, 4]. Genomes have been shaped and restructured dynamically. Related application includes [5] annotation of newly sequenced genomes [6], identification of conserved noncoding sequences [7], estimation of whole genome duplication events [1], prediction of chromosomal rearrangements, and the structure of ancestral genomes [8]. As a result, the procedure of synteny and collinearity analysis has become a hot topic in evolutionary biology as a standard step for elucidating the evolutionary histories of both genomes and gene families.

To meet the requirement of synteny and collinearity analysis, the majority of softwares focus on the detection and alignment of the original sequencing data. Using the traditional clustering of neighboring match of gene pairs, various softwares have been developed to match gene pairs, including ADHoRe [9], the Max-gap Clusters by Multiple Sequence Comparison (MCMuSeC) [10], and OrthoCluster [11, 12]. More recent methods apply dynamic algorithm to pairwise collinear genes chains, in which a matching system scores the adjacent collinear gene pairs, known as anchor genes, and penalizes the distance between anchor genes. This method has been implemented in software tools such as ColinearScan [13], MCScan [1], SyMAP [6], FISH [14], and CYNTENATOR [15]. Besides the pairwise collinear relationships among chromosomal regions, the multialignment (alignment of three or more regions) of collinear chromosomal regions (referred to as collinear blocks) is more important as it can reveal ancient WGD events [1] and complex chromosomal duplication/rearrangement relationships [16]. One of the early software packages providing analysis of collinearity within gene families is MicroSyn [17]. MCScan [1], Multiple Collinearity Scan, is another very popular algorithm in synteny and collinearity detection. It scans multiple genomes or subgenomes, identifies putative homologous chromosomal regions, and marks these gene regions with alignment anchors. The latest i-ADHoRe 3.0 [18] combines pairwise comparison with an iterative profile search, and it uses rigorous statistical tests to ensure that regions found are significant. All these software packages have focused on the process of data rather than downstream analysis. Many of them do not even provide visual graphic outputs.

Another class of synteny and collinearity tools works with the general-purpose genome browsers, which are softwares that allow the user to view genome annotations in the context of a reference sequence. Most of them use vector graphics to enable the scrolling and zooming through arbitrary regions of a genome. GBrowse-syn [19] is the plugin of GBrowse 2.0 [20, 21], one of the most powerful web-based applications to visualize genomic data. It allows the comparison of collinear regions of multiple genomes using the GBrowse-styled web page, in which the synteny and collinearity are displayed as traditional connection diagram. This kind of general-purpose software packages however only provides very basic drawings, as they are not designed to meet the advance visualization requirement of the synteny and collinearity representation.

As synteny and collinearity visualization becomes increasingly important, many specific software programs have been developed lately. Most of these software programs, such as SynChro [5], GSV [22], and Easyfig [23], however inherit the linear tradition in this area, which plots the synteny and collinearity relationship into lines and bars. A typical output style uses two bars for the chromosomes and lines for the colocational relationship. While it is easier and more convenient to use web based interface to generate the linear plot, it is difficult for research reporting, especially for paper pipelines. The extension package of MCScan named MCScanX [24] implements 15 utility programs for display and analyses. However, MCScanX provides a command-line based plotter with PNG output only. Another case in point is that the i-ADHoRe 3.0 [18] extends ADHoRe [9] and provides a package to draw dot plot in SVG vector graphics and PNG raster images.

Circos [25] is a well-known visualization tool using circular ideogram layout to facilitate the identification and analysis of similarities and differences found in comparisons of genomes. Raster or vector images can be created from GFF-style data inputs and hierarchical configuration files, which are popular in bioinformatics researches, making Circos suitable for rapid reporting pipelines. A typical case is C-Sibelia [26], which focuses on the synteny and collinearity analysis and outputs the Circos-formated file to plot. Many recent genetic research reports in Nature and Science have applied Circos-styled figures, but still it only provides circular plot.

There are many online platforms for genome evolution that are dedicated to synteny and collinearity analysis. Meanwhile, more and more researchers use their visualization services in their research procedures. Since the cost of calculation grows exponentially with the amount of data, particularly in the process of analysis, most of these platforms provide dotted or linear plot because it is much simpler and faster to accomplish. Examples of such platforms include Plant Genome Duplication DataBase [27], MIPS CrowsNest [28], and Yeast Gene Order Browser [29]. Only very few platforms can generate complex plots, such as circular plot and multialignment plot, for example, the famous Ensembl [30, 31]. In plant comparative genomics, PLAZA 3.0 is one of the most powerful all-in-one solutions in this area. It has collected a large quantity of data and developed the full utility sets to support research from analyses to visualizations [32]. And yet none of them provides full support of vector graphic outputs. The gap for multistyled vector-based plots in synteny and collinearity remains to be filled.

Generally, synteny and collinearity analysis is a frequent task in comparative genomics research. Many analysis software packages are available, but not effective in visualizing the result, shown in Table 1. The problems include lack of graphic visualization, simple representation, or inextensible output format. On the other hand, general-purpose visualization tools are powerful, but not specific for synteny and collinearity display. This requirement grows rapidly while higher throughput of datasets generates higher resolution outputs.

In this paper, we introduce VGSC, a purpose-built toolkit in visualizing the synteny and collinearity into general graphical format, including both raster (JPEG, Bitmap, and PNG) and vector graphics (SVG, EPS, and PDF).

Vector graphics are a computational representation of graphical objects using vectors, a geometric object with a magnitude and a direction. In this way, vector graphics are normally combinations of geometrical primitives, such as points, lines, curves, shapes, and polygons. In contrast, raster images use dot matrix data to represent a generally rectangular grid of pixels or points of color. The advantages of vectors are scale-invariance, rotate-invariance, and transform-invariance. They enable the antialiasing feature, which means graphics can be magnified infinitely without loss of quality. Therefore, vector graphics are widely used in scientific research, especially in the bioinformatics research where a massive amount of data from the sequencing process generates various types of high-resolution graphs. A good case in point is WebLogo [33], which is a software package to generate sequence logos, the graphical representations of the patterns within a multiple sequence alignment. WebLogo is so popular that in some areas it becomes the gold standard. This tool is very effective and efficient because it provides both command line interface and web interface, as well as both raster and vector graphics as outputs.

## 2. Implementation and Result

### 2.1. Software Architecture

**Figure 1:**
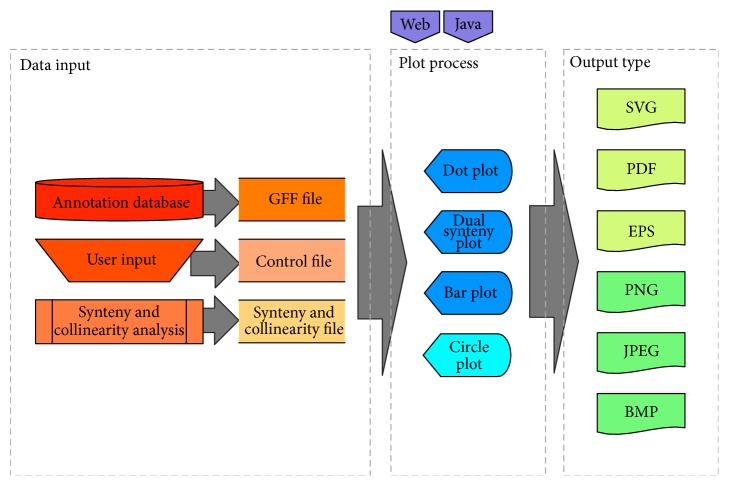
System architecture of VGSC.

Vector Graphic toolkit of genome Synteny and Collinearity (VGSC) is a new web-based interface for synteny and collinearity representation. Its software architecture is shown in Figure 1, in which the command-line toolkits and web-based service are both illustrated. The workflow of plotting remains as simple as most visualization tools: the end user prepares the required datasets and configures the basic parameters; the software then plots accordingly. Many of these features have simplified the process of drawing, so that researches can focus more on the analysis and interpretation of the data.

### 2.2. Data Input and Configuration

**Figure 2:**
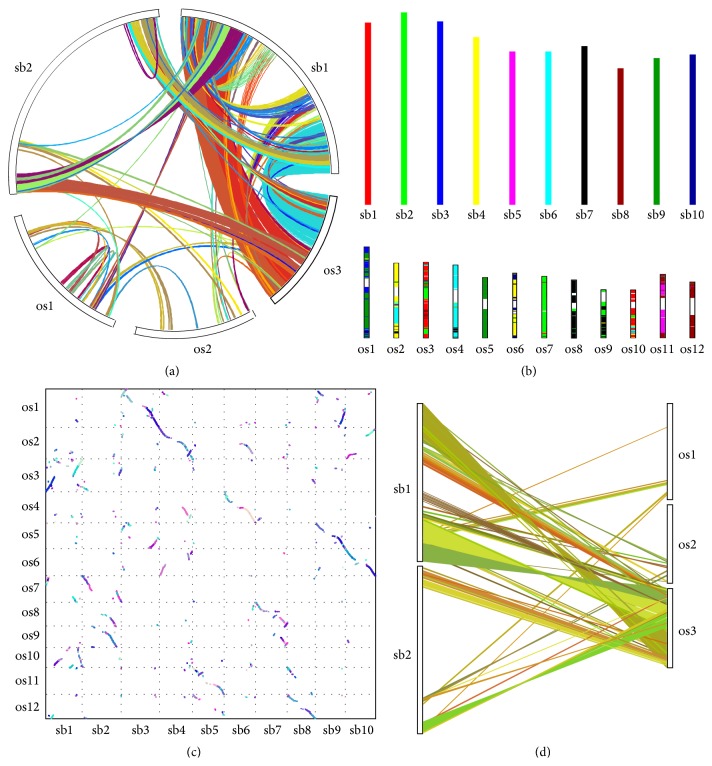
Four types of synteny and collinearity plot: (a) Circle Plot, (b) Bar Plot, (c) Dot Plot, and (d) Dual Synteny Plot. Chromosomes are labeled in species abbreviation plus chromosome ID. os,* Oryza sativa*; sb,* Sorghum bicolor*.

In Figure 2, three inputs from end users are required: (1) synteny and collinearity file, (2) gene annotation file, and (3) control file. And they are explained as follows:

1. *Synteny and collinearity file*: VGSC operates on the preprocessed synteny and collinearity data. It is easy to convert results from all the common synteny and collinearity analysis software packages into the required format. The detailed requirement is available in the software manual.
2. *Gene annotation file*: this GFF3 annotation file (http://www.gmod.org/wiki/GFF3) provides the fundamental map for the plotting, which is widely used in gene assembling software and gene databases.
3. *Control file*: in this file, the detailed configuration sets the width, length, color, and so forth for the plot.

If end users run VGSC in the command line, these settings serve as inputs as textual parameters. A Java Runtime Environment 1.8 is mandatory, as the software is packaged as a Java executable. For users, synteny and collinearity file and annotation file should be uploaded, and the parameters in the control file can be configured directly in the web form. In addition, we have listed a set of data samples with preconfigured parameters in the “Example” section of the website to help end users carry out tests.

### 2.3. Output and Result

VGSC provides four different types of plots in six different file formats, with which the synteny and collinearity information can be drawn into circle, bars, dots, and dual synteny. Figure 2 demonstrates the four plots generated by a sample data set of the synteny and collinearity across Rice (*Oryza sativa*) and Sorghum (*Sorghum bicolor*) from MCScanX website (http://chibba.pgml.uga.edu/mcscan2).

In the command-line executable, we have implemented a plot manager to integrate all types of plots into one command, which has made the selection much easier. We have introduced a multiple file format adaptor, which enables both raster and vector graphics, so that the output file formats expend to SVG, EPS, PDF, JPEG, and BMP, in addition to the popular PNG format. This automatic configuration mechanism is also applied to all the parameter settings, and the detailed settings list is in the software manual, available at http://bio.njfu.edu.cn:8080/vgsc-web/static/downloads/vgsc-manual.pdf.

**Figure 3:**
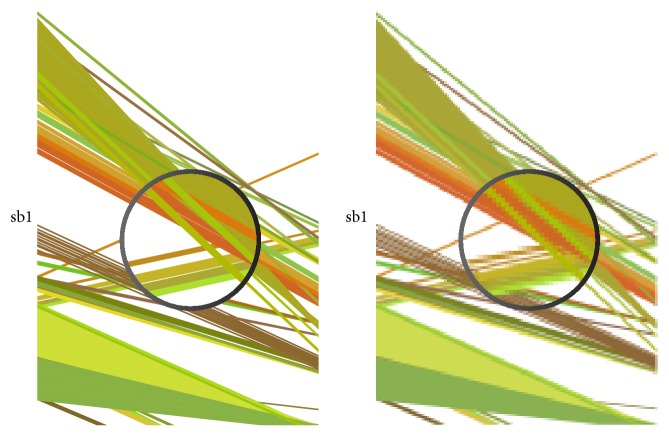
Resolution comparison between vector graphics and raster graphics.

One of the most important features of VGSC is its ability to produce vector graphics. As Figure 3 demonstrates, compared with raster graphics (right), vector graphics (left) provide higher compatibility when the image is magnified. This is particularly noticeable when high-throughput datasets are concerned. High-quality images are often a requirement for scientific research reports and papers.

For web users, there is a list of options, where end users can specify the type of plot. A dropdown menu is also available, where end users can choose the output file format. Once the settings are confirmed, results can be downloaded as a separate file when the “Download” button is clicked. In the online service, both vector graphics and raster images are provided.

### 2.4. Online System

**Figure 4:**
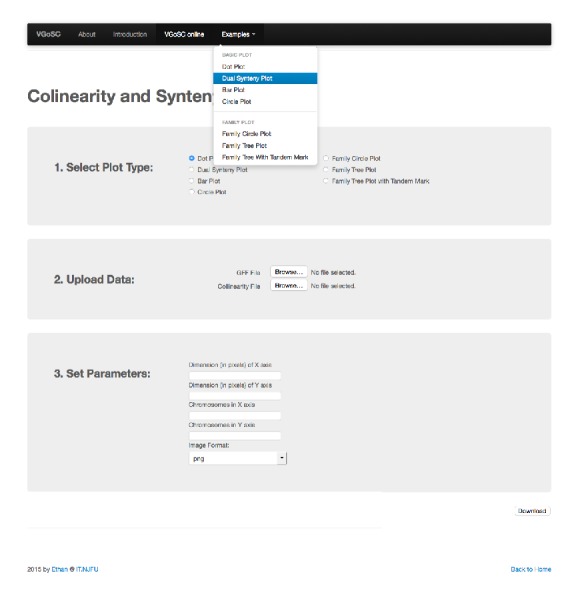
Screenshot of VGSC online.

Parallel with command-line toolkit, we have published a web-based system, VGSC online, to provide the plotting service and to improve the experience in plotting. It is available at http://bio.njfu.edu.cn:8080/vgsc-web. The VGSC online uses Java Web Technology and is compatible with most of web containers including Tomcat and jetty.  Figure 4 shows the screenshot from the example pages in VGSC online. It lists all types of plots with sample data, providing end users with a visual scaffold. We have also published the command line executable for downloading, along with some sample data and relevant documentation. All these resources are provided free.

## 3. Conclusion

While many synteny and collinearity tools have become available in recent years, their visual presentation has not been developed accordingly. For this reason, users often have to write additional programs or redraw the synteny and collinearity output files in order to plot a representative high-quality image. This incompleteness of visualization has reduced the efficiency of existing synteny and collinearity detection pipeline. VGSC has been created to fill this gap. A distinguishing feature of VGSC and its online service is that diverse tools for vector graphics of synteny and collinearity are incorporated, which enables rapid and convenient conversion of synteny and collinearity information into graphical insights. Additional plots for downstream analysis, such as plots for gene family, will be implemented in the coming version of VGSC. VGSC therefore will also be an effective tool for structural changes and evolution analysis, annotation for new genomes, and gene family history research.

## Figures and Tables

**Table 1:** Software list for synteny and collinearity visualization.

Software name	Publishing year	Graphical synteny	Visualization types	Vector graphics
MCMuSeC	2009	*✕*	*✕*	*✕*
OrthoCluster	2009	✓	Dual bar, linear	*✕*
i-ADHoRe 3	2011	✓	Dotted hierarchy	**✓**
FISH	2003	*✕*	*✕*	*✕*
ColinearScan	2006	*✕*	*✕*	*✕*
MCScan	2008	*✕*	*✕*	*✕*
CYNTENATOR	2010	*✕*	*✕*	*✕*
SyMAP	2011	✓	Dual bar	*✕*
MCScanX	2012	✓	**Dotted linear circular**	*✕*
SynChro	2014	✓	Dual bar	*✕*
GSV	2011	✓	Dual bar	*✕*
EasyFig	2011	✓	Dual bar	**✓**
C-Sibelia	2013	*✕*	Circos-format	*✕*
Gbrowse-syn	2010	✓	Dual bar	*✕*
Kegg	2000	✓	Network	*✕*
Circos	2009	✓	Circular	✓
WebLogo	2004	*✕*	Textual	✓
**VGSC**	**2015**	**✓**	**Dotted linear circular**	**✓**
