# Corrigendum to “VGSC: A Web-Based Vector Graph Toolkit of Genome Synteny and Collinearity”

**DOI:** 10.1155/2016/4948583

**Published:** 2016-09-14

**Authors:** Yiqing Xu, Changwei Bi, Guoxin Wu, Suyun Wei, Xiaogang Dai, Tongming Yin, Ning Ye

**Affiliations:** ^1^School of Computer Science and Engineering, Southeast University, Nanjing, Jiangsu 211189, China; ^2^The Southern Modern Forestry Collaborative Innovation Center, Nanjing Forestry University, Nanjing, Jiangsu 210037, China; ^3^College of Information Science and Technology, Nanjing Forestry University, Nanjing, Jiangsu 210037, China; ^4^College of Forest Resources and Environment, Nanjing Forestry University, Nanjing, Jiangsu 210037, China

In the article titled “VGSC: A Web-Based Vector Graph Toolkit of Genome Synteny and Collinearity” [1], in Section 2.3. (Output and Result) this URL http://bio.njfu.edu.cn:8080/vgsc-web/static/downloads/vgsc-manual.pdf should be replaced by this URL http://bio.njfu.edu.cn/vgsc-web/static/downloads/vgsc-manual.pdf and in Section 2.4. (Online System), this URL http://bio.njfu.edu.cn:8080/vgsc-web should be replaced by this URL http://bio.njfu.edu.cn/vgsc-web.
