# Corrigendum #2 to “VGSC: A Web-Based Vector Graph Toolkit of Genome Synteny and Collinearity”

**DOI:** 10.1155/2019/2150291

**Published:** 2019-05-30

**Authors:** Yiqing Xu, Changwei Bi, Guoxin Wu, Suyun Wei, Xiaogang Dai, Tongming Yin, Ning Ye

**Affiliations:** ^1^School of Computer Science and Engineering, Southeast University, Nanjing, Jiangsu 211189, China; ^2^The Southern Modern Forestry Collaborative Innovation Center, Nanjing Forestry University, Nanjing, Jiangsu 210037, China; ^3^College of Information Science and Technology, Nanjing Forestry University, Nanjing, Jiangsu 210037, China; ^4^College of Forest Resources and Environment, Nanjing Forestry University, Nanjing, Jiangsu 210037, China

The article titled “VGSC: A Web-Based Vector Graph Toolkit of Genome Synteny and Collinearity” [1] was found to contain text, figures, and software similarities with another published article cited as reference #24 in the reference list [2]. The authors used that software, MCScanX, as the basis of the data and figures in the article, without clearly attributing their use of this program.

The authors reimplemented the MCScanX software and extended the utility of MCScanX in two aspects: first, VGSC is able to reproduce MCScanX-generated images as vector graphics, with which researchers can obtain scale-independent and direction-independent versions of MCScanX-generated images for inclusion in published articles; second, MCScanX uses command-line terminals to perform calculations and visualization, whereas VGSC provides an online avenue to utilize MCScanX, vgsc-web.

The authors apologize for not clearly attributing their use of the MCScanX software and note that any study that utilizes VGSC needs to also reference MCScanX's original publication [2].

In addition, Dr. Ning Ye was incorrectly listed as the corresponding author. The corresponding author is Dr. Yiqing Xu.
